# Supplementary material for: Identification and functional analysis of non-coding regulatory small RNA FenSr3 in Bacillus amyloliquefaciens LPB-18
Source: PeerJ. 2023 May 15;11:e15236. doi: 10.7717/peerj.15236 (PMC10194069; doi:10.7717/peerj.15236)
Supplement: Supplemental Information 4 [file peerj-11-15236-s004.zip › KO/CK-vs-T1_map/map05142.html]

KEGG PATHWAY: Chagas disease (American trypanosomiasis) - Reference pathway


|  |  |
| --- | --- |
| **Chagas disease (American trypanosomiasis) - Reference pathway** |  |

[
Pathway menu
| Organism menu
| Pathway entry
| Show description
| User data mapping
]

Trypanosoma cruzi is an intracellular protozoan parasite that causes Chagas disease. The parasite life cycle involves hematophagous reduviid bugs as vectors. Once parasites enter the host body, they invade diverse host cells including cardiomyocytes. Establishment of infection depends on various parasite molecules such as cruzipain, oligopeptidase B, and trans-sialidase that activate Ca2+ signaling. Internalized parasites escape from the parasitophorous vacuole using secreted pore-forming TcTOX molecule and replicate in the cytosol. Multiplied parasites eventually lyse infected host cells and are released in the circulation. During these events, the parasites manipulate host innate immunity and elicit cardiomyocyte hypertrophy. T lymphocyte responses are also disturbed.

|  |  |  |
| --- | --- | --- |
| Reference pathway | 184% 150% 122% 100% 82% 67% 55% | 图片下载 |
